# From Physical Replacement to Biological Symbiosis: Evolutionary Paradigms and Future Prospects of Auditory Reconstruction Brain–Computer Interfaces

**DOI:** 10.3390/mi17030343

**Published:** 2026-03-11

**Authors:** Li Shang, Juntao Liu, Shiya Lv, Longhui Jiang, Yu Liu, Sihan Hua, Jinping Luo, Xinxia Cai

**Affiliations:** 1 State Key Laboratory of Transducer Technology, Aerospace Information Research Institute, Chinese Academy of Sciences, Beijing 100190, China; shangli23@mails.ucas.ac.cn (L.S.); lvshiya21@mails.ucas.ac.cn (S.L.); jianglonghui22@mails.ucas.ac.cn (L.J.); liuyu222@mails.ucas.ac.cn (Y.L.); huasihan24@mails.ucas.ac.cn (S.H.); 2 University of Chinese Academy of Sciences, Beijing 100049, China

**Keywords:** auditory brainstem implant, neural engineering, bio-integration, neural encoding, deep learning, neuromodulation, auditory cortex

## Abstract

Auditory Brain–Computer Interfaces (BCIs) constitute the vital intervention for profound sensorineural hearing loss where the auditory nerve is compromised, yet their clinical efficacy remains restricted by substantial biological bottlenecks and limited spectral resolution. This review critically examines the evolutionary paradigm of auditory restoration, tracing the transition from static physical replacement to dynamic biological symbiosis. We systematically analyze physiological barriers across cochlear, brainstem, and cortical levels, elucidating how rigid interfaces provoke chronic tissue responses and why linear encoding protocols fail in distorted central tonotopy. The article synthesizes emerging methodologies in material science, demonstrating how soft, bio-integrated electronics and biomimetic topologies effectively address mechanical impedance mismatches. Furthermore, the trajectory of neural encoding is evaluated, highlighting the paradigm shift from traditional envelope extraction to deep learning-driven non-linear mapping and adaptive closed-loop neuromodulation. Finally, the potential of high-resolution modulation techniques, including optogenetics and sonogenetics, alongside AI-facilitated intent perception for active listening, is assessed. It is concluded that future neuroprostheses must evolve into symbiotic systems capable of seamlessly integrating with neural plasticity to enable high-fidelity cognitive reconstruction.

## 1. Introduction

### 1.1. Auditory Impairment and Societal Needs

Audition serves not merely as the fundamental sensory modality for perceiving the physical environment, but effectively functions as the core nexus facilitating language acquisition, emotional interaction, and social engagement. Under normal physiological conditions, auditory perception relies on a precise ascending neural pathway: sound waves are mechanically transmitted via the outer and middle ear to the cochlea, driving the vibration of the basilar membrane and activating inner hair cells to transduce mechanical energy into neural electrical impulses [1] (pp. 3–25). Subsequently, these signals are transmitted by spiral ganglion neurons via the auditory nerve, ascending sequentially through the cochlear nucleus, the superior olivary complex, the midbrain inferior colliculus, and the thalamic medial geniculate body, before finally projecting to the auditory cortex for complex spectro-temporal decoding and semantic reconstruction. However, interruption at any stage of this cascade—whether due to peripheral hair cell damage or abnormalities in central neural transmission—can result in sensorineural hearing loss (SNHL), effectively impeding the input of information to the brain.

Currently, hearing loss represents a severe challenge in the field of global public health. According to the World Report on Hearing published by the World Health Organization (WHO), over 430 million people globally are currently afflicted by disabling hearing loss. Driven by factors such as an aging population and increased environmental noise exposure, this figure is projected to surge to over 700 million by 2050, implying that one in every ten individuals will face the threat of silence [2]. More critically, the consequences of auditory deprivation extend far beyond the sensory dimension. According to the latest comprehensive analysis by the Lancet Commission regarding dementia intervention, hearing impairment during midlife emerges as the paramount modifiable predictor for late-onset dementia, with a population attributable fraction (PAF) estimated at 8% [3]. Chronic deprivation of auditory input induces maladaptive plastic changes in the brain, which not only significantly increases the risks of depression and social isolation but also accelerates the depletion of cognitive reserve, thereby imposing a dual burden on individuals’ quality of life and healthcare systems [3,4].

Regarding clinical intervention, although the cochlear implant (CI) has established itself as the gold standard for treating severe-to-profound sensorineural hearing loss, its efficacy is strictly contingent upon the anatomical integrity of the auditory nerve. For patient populations unable to benefit from conventional CIs—such as those with bilateral auditory nerve damage due to Neurofibromatosis Type 2 (NF2), severe cochlear ossification, or congenital cochlear nerve aplasia—traditional technologies not only fail to restore hearing but may also further damage residual structures due to surgical trauma [5]. This “therapeutic blind spot”, unreachable by existing medical means, urgently necessitates a neuroprosthetic technology capable of bypassing compromised peripheral organs to interact directly with the central auditory pathway (such as the brainstem or midbrain) [6]. This necessity constitutes the fundamental logic and clinical impetus for the development of auditory reconstruction brain–computer interfaces.

### 1.2. Evolution Paradigm of Auditory Reconstruction Technology

In the face of severe-to-profound sensorineural hearing loss, where traditional hearing aids frequently yield negligible efficacy, there has been a compelling imperative for implantable technologies capable of direct interaction with the nervous system. It is against this backdrop that the auditory reconstruction BCI has emerged as a vanguard neuroprosthetic technology. Fundamentally, this technology is defined by its ability to bypass compromised peripheral auditory organs—principally the cochlea—and deliver precisely modulated electrical stimulation directly to the central auditory pathway (e.g., the cochlear nucleus, inferior colliculus, or auditory cortex) via implanted electrode arrays, thereby “reconstructing” auditory perception within the brain [7].

Retrospectively, the genesis of auditory BCIs can be traced to the mid-20th century with the early elucidation of electrophonic phenomena, which substantiated that electrical stimulation of the auditory pathway could elicit sound perception. By the 1970s and 1980s, the successful development and clinical dissemination of the first-generation cochlear implant marked the transition of the nascent auditory BCI from conceptualization to reality, establishing the foundational paradigm of “hearing reconstruction via electrical stimulation” [8,9]. Subsequently, to address the needs of patients for whom cochlear implantation was contraindicated due to pathologies such as acoustic neuroma, the Auditory Brainstem Implant (ABI) was advanced during the 1990s and early 2000s, elevating the intervention target for the first time from the periphery to the brainstem level [5,10]. Currently, the research frontier has shifted focus toward Auditory Midbrain Implants (AMI) and Auditory Cortical Interfaces (ACI), dedicated to overcoming the critical challenge of perceptual naturalness through the deployment of superior interface technologies and encoding strategies [11].

### 1.3. Outline of This Review

This review aims to establish a systematic framework spanning from underlying physiological mechanisms to top-level technological implementation. Section 2 begins with a bottom-up dissection of the specific physiological barriers encountered by interfaces at varying levels—from the peripheral cochlea and brainstem nuclei to the central auditory cortex. It specifically elaborates on the profound constraints imposed on reconstruction outcomes by maladaptive neural plasticity induced by auditory deprivation (such as tinnitus). In response to these biological constraints, Section 3 focuses on the physical architecture of neural interfaces, discussing how strategies involving the flexible modification of advanced materials, biomimetic topological design, and adaptive minimally invasive implantation address the mechanical impedance mismatch between abiotic devices and biological tissues to achieve long-term, stable, and highly biocompatible integration. Section 4 delves into the core of information interaction—neural encoding strategies—analyzing the algorithmic evolution from traditional envelope extraction to high-fidelity signal inscription based on physiological models. Particular emphasis is placed on the critical role of deep learning-driven non-linear mapping and closed-loop control in resolving the complex topological encoding required by the central nervous system. Finally, Section 5 forecasts fundamental paradigm shifts in the field, ranging from high-resolution optogenetic modulation and bio-electronic symbiotic development to intent-aware intelligent ecosystems, while also scrutinizing the neuroethical and social equity challenges accompanying such deep technological intervention.

Through this progressive analysis, this article strives to provide readers with a macroscopic perspective that transcends the limitations of singular implantation targets. We aim not only to reveal the intrinsic logic driving the evolution of auditory reconstruction technology—from early “physical electrode stacking” to “synergistic soft-interface and intelligent modulation”—but also to establish an interdisciplinary conceptual framework bridging basic neuroscience, materials engineering, and artificial intelligence. Ultimately, we aspire to provide prospective theoretical references and practical insights for addressing future high-order clinical demands, facilitating the transition from simple sound perception restoration to high-fidelity, intent-driven auditory cognitive reconstruction.

## 2. Advantages and Bottlenecks of Interfaces at Various Levels of the Auditory Pathway

**Figure 1:**
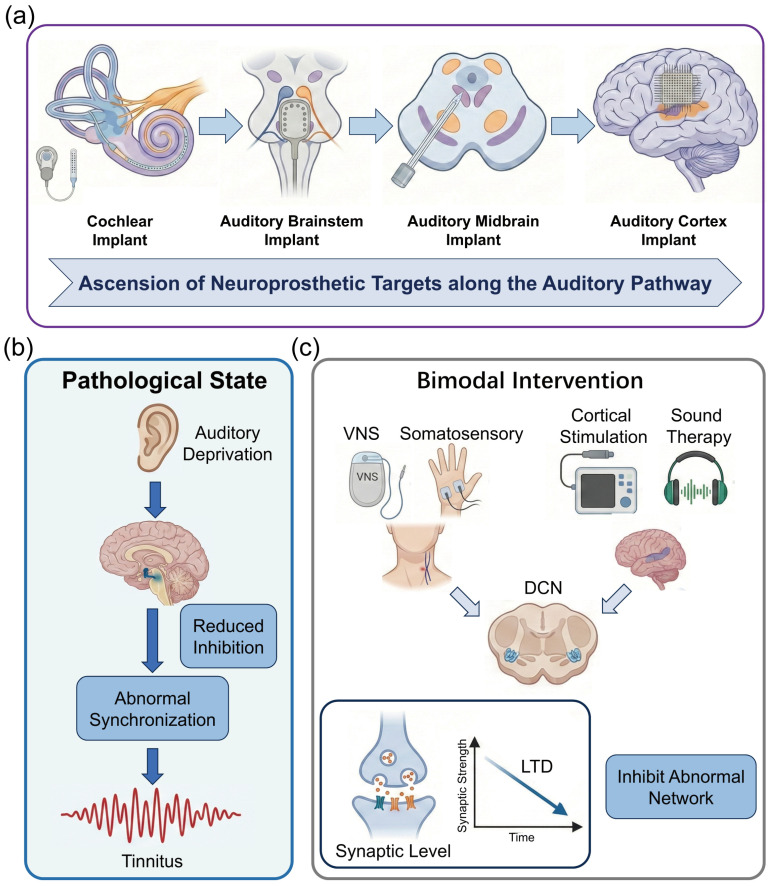
Anatomical hierarchy of auditory interfaces and mechanisms of deprivation-induced plasticity and neuromodulation. (**a**) Schematic illustration of the ascending auditory pathway and stratified neuroprosthetic intervention targets. (**b**) Mechanisms of maladaptive neuroplasticity induced by auditory deprivation. (**c**) Therapeutic neuromodulation strategies and underlying mechanisms. (created with https://scidraw.io, CC BY 4.0).

The hierarchical architecture of the auditory pathway inherently dictates the diversity and complexity of neuroprosthetic intervention strategies. From the peripheral cochlea to the central cortex, each level of neural interface presents unique therapeutic advantages and specific technical bottlenecks when reconstructing auditory function. Peripheral interfaces, predicated on a relatively well-defined tonotopy, are capable of delivering mature auditory input; however, their efficacy is strictly constrained by the integrity of the residual auditory nerve. Conversely, as intervention targets ascend toward the central nervous system (including the brainstem, midbrain, and cortex) (Figure 1a), while offering the potential to bypass severe peripheral pathologies and directly facilitate cognitive reconstruction, they inevitably confront more intricate anatomical distortions and biophysical challenges. This chapter will systematically dissect the anatomical characteristics of targets at each level, dialectically evaluating their functional potential in clinical applications alongside the limitations of extant technologies. Furthermore, it will expound upon the feasibility of integrating neuromodulation strategies to mitigate comorbid pathological alterations (such as tinnitus), thereby furnishing a theoretical groundwork for choosing the best intervention routes.

### 2.1. Transmission Limitations of Cochlear Interfaces

The developmental history of the Cochlear Implant stands as one of the most significant clinical translational achievements in the field of neural engineering. The genesis of this technology can be traced back to 1957, when Djourno and Eyriès first successfully induced high-frequency environmental sound perception by directly stimulating the auditory nerve of a totally deaf patient with implanted electrodes, thereby verifying the feasibility of “electric hearing” [12,13]. Subsequently, although the single-channel implant system developed by House et al. was limited by its information transmission capacity and could only facilitate lip-reading, it laid the foundation for subsequent research [8]. The true technological leap occurred in the 1980s, when teams led by Graeme Clark and Ingeborg Hochmair, respectively, developed multi-channel electrode arrays. In conjunction with the Continuous Interleaved Sampling (CIS) method established by Wilson et al. (1991), this effectively resolved the issue of signal interference inherent in multi-channel stimulation [14]. This series of technological innovations elevated sentence recognition rates in quiet environments for patients with severe sensorineural hearing loss from less than 10% to over 80%, establishing the CI as the clinical gold standard for auditory reconstruction [15].

Despite the immense success of modern cochlear implants in speech recognition, they fundamentally remain low-resolution neural prostheses [16]. First, the insufficiency of spectral resolution constitutes the primary technical bottleneck at present. Whereas a normal cochlea utilizes approximately 3500 inner hair cells to achieve precise frequency encoding [17], commercial CI systems typically possess only 12 to 24 physical electrode contacts. More critically, as the scala tympani is filled with highly conductive perilymph, the current released by electrode contacts is not confined to local neurons but generates extensive current spread [18]. This physical phenomenon results in severe channel interaction, where electric fields generated by adjacent electrodes overlap spatially [19]. A classic study by Friesen et al. (2001) confirmed through psychophysical experiments that even if an implant possesses a higher number of physical electrodes, the number of independent information channels (Effective Channels) actually available to the patient’s brain typically does not exceed eight [20]. This “bottleneck effect” explains why existing technology is sufficient to support coarse speech envelope recognition but performs poorly in scenarios requiring fine spectral information, such as speech recognition in noise or music appreciation.

In addition to spectral resolution, the absence of Temporal Fine Structure (TFS) further limits the quality of auditory perception. Current sound coding strategies predominantly extract the amplitude envelope information of sound while discarding the fine temporal information that carries pitch and timbre characteristics. Research by Zeng et al. (2005) indicated that a lack of TFS information makes it difficult for patients to perceive changes in fundamental frequency (F0), causing significant difficulties for CI users in processing tonal languages (such as Mandarin Chinese) or appreciating melodies [21]. Although researchers have attempted to ameliorate this issue by increasing stimulation rates or employing virtual channel technology, the limitations imposed by the refractory period and synchronization capabilities of neurons mean that optimization via coding strategies alone cannot fully compensate for information loss at the neural interface hardware level.

Furthermore, mechanical trauma during electrode insertion and long-term tissue response constitute another anatomical dilemma. The human cochlea is a delicate spiral structure, and the process of inserting an electrode array into the scala tympani is prone to causing mechanical damage to microstructures such as the basilar membrane and spiral ligament. Histopathological analysis by Holden et al. (2013) pointed out that fibrotic tissue proliferation induced by implantation trauma can encapsulate the electrode; this not only leads to the loss of residual hearing but also increases electrode impedance, necessitating higher current intensity from the system, which in turn exacerbates the range of current spread [22]. To optimize the coupling efficiency of the neural interface, Skinner et al. (2002) evaluated the clinical efficacy of the pre-curved electrode (Perimodiolar Electrode) in detail, attempting to lower stimulation thresholds by positioning the electrode snugly against the modiolus [23]. However, such designs increase the surgical risk of the electrode penetrating the basilar membrane and entering the scala media or scala vestibuli; once electrode translocation occurs, it severely disrupts the ionic homeostasis and neural transmission environment within the cochlea. Therefore, achieving minimally invasive implantation while ensuring stimulation precision remains an urgent challenge to be resolved in the field of cochlear interfaces.

### 2.2. Structural Challenges of Brainstem Nucleus Implantation

Although engineering approaches have attempted to enhance the resolution of cochlear interfaces through current focusing techniques, peripheral intervention lacks an anatomical basis in patients with severe auditory nerve damage or aplasia. To reconstruct the auditory pathway, the intervention target must bypass the cochlea and directly engage the anatomically more complex brainstem centers; this presents a fundamental challenge for auditory reconstruction, necessitating a paradigm shift from peripheral nerve stimulation to central neuromodulation.

When patients are unable to benefit from cochlear implants due to bilateral auditory nerve compromise (e.g., NF2), cochlear nerve aplasia, or severe cochlear ossification, the ABI represents the sole viable modality for reconstructing the auditory pathway. Its core rationale lies in completely bypassing the compromised cochlea and auditory nerve by placing stimulation electrodes directly onto the surface of the cochlear nucleus within the cerebellopontine angle (CPA) [5,24,25]. As the first-order neuronal convergence point of the central auditory system, the cochlear nucleus is responsible not only for preliminary frequency analysis but also for the critical function of transmitting auditory signals to higher centers [26]. Globally, the clinical application of ABIs exhibits significant population-specific variability. Large-scale clinical studies by Sennaroglu et al. indicate that non-tumor patients with congenital cochlear nerve aplasia or cochlear ossification typically achieve superior auditory perception and speech recognition compared to NF2 patients suffering from anatomical distortion due to tumor compression [27]. Although ABIs facilitate fundamental sound perception, speech recognition rates in the majority of patients remain significantly below expectations; in particular, open-set speech comprehension in the NF2 patient population continues to be markedly deficient [28].

Existing commercial ABI devices (e.g., Cochlear, Med-El) continue to utilize rigid planar electrode architectures designed decades ago, resulting in severe hardware performance bottlenecks. First, spectral resolution is critically low. Unlike the linear regularity of the cochlear basilar membrane, the tonotopic organization of the cochlear nucleus surface is complex; current electrodes offer only approximately 12 to 21 low-density physical contacts with relatively large surface areas. This results in highly diffuse spatial stimulation signals, preventing the realization of precise frequency encoding [29]. Second, suboptimal biocompatibility leads to a decline in long-term efficacy. The mechanical mismatch between the rigid substrate and soft brain tissue induces a sustained immune response, generating a fibrotic capsule up to several hundred micrometers thick; this significantly increases electrode impedance and further deteriorates stimulation precision.

At the level of clinical implementation, anatomical risks and non-auditory side effects constitute the core difficulties limiting the widespread adoption of ABIs. The cochlear nucleus target is deeply embedded within the CPA, in close proximity to the facial, vestibular, and vagus nerve nuclei. As existing electrodes cannot achieve conformal contact with the brainstem surface, the risk of electrode migration is extremely high [30]. More critically, electrical current is prone to spilling over into adjacent nuclei, triggering severe non-auditory side effects such as facial twitching, vertigo, or a sensation of a foreign body in the throat. Consequently, clinicians are compelled to deactivate as many as 30% to 50% of critical channels during fitting, thereby drastically compressing the transmission bandwidth for auditory information [31,32].

Addressing these dilemmas, the team led by Stephanie P. Lacour has leveraged advanced nanofabrication techniques and flexible electronics to develop an ultra-flexible neural interface with high biocompatibility [33]. This interface not only boasts a contact density several times higher than existing commercial devices but also achieves seamless conformal contact with the brainstem surface. The superior conformability of this soft, 150-μm-thick silicone-based array, governed by an elastocapillarity-driven design, allows it to wrap around the complex curvilinear topography of the cochlear nucleus. By minimizing the electrode-to-neuron distance and reducing the resistive drop at the interface, this novel flexible interface significantly reduces stimulation thresholds in vivo, with activation thresholds measured as low as 0.3 mA. Furthermore, high spatial resolution is achieved through the use of microfabricated electrodes with small diameters (100 μm), which facilitate high local charge density and focal activation of specific neuronal populations. This precision effectively confines stimulation within auditory sub-regions and circumvents non-auditory side effects, such as facial twitching or pain, typically caused by unintended current spread in macroscopic clinical implants. This research signifies a pivotal advancement for ABIs from “coarse current activation” toward “precise encoding”, providing a promising solution to the persistent clinical problem of low spectral resolution. However, despite these advancements, several key challenges remain for clinical translation. While the device maintains stable electrochemical properties, a progressive increase in activation thresholds was observed over time, potentially due to micromotion-induced foreign body reactions. Notably, experimental evidence from macaque models also highlighted the risk of electrode migration; without robust surgical fixation, the ultra-soft array may shift relative to the target nucleus, leading to a loss of spatial tuning and spectral resolution. Additionally, thin-film devices remain more vulnerable to water ingress and metal corrosion compared to bulk macroscopic devices, necessitating the future development of advanced hermetic packaging to ensure multi-year reliability in human patients.

### 2.3. Signal Relay and Cognitive Reconstruction Mechanisms in the Central Auditory System

When lesions of the auditory pathway involve the cochlear nucleus (e.g., following severe brainstem stroke or tumor resection), or when modulation of higher-order pathological auditory networks (such as intractable tinnitus) is required, the implantation target must be advanced superiorly to the midbrain or cortex. Interfaces at this level function not merely as “afferent channels” for acoustic information but, more critically, as “reconstruction hubs” for auditory cognition and neuromodulation.

#### 2.3.1. Auditory Midbrain Implant Strategies

The Inferior Colliculus (IC) constitutes an obligatory relay nucleus in the auditory pathway, serving as a convergence point for virtually all ascending fibers from the brainstem. In contrast to the complex, non-linear frequency distribution characteristic of the cochlear nucleus surface, the Central Nucleus of the Inferior Colliculus (ICC) exhibits a highly ordered, three-dimensional laminar tonotopy. Electrophysiological mapping studies by Lim and Anderson (2007) revealed that the ICC is composed of a series of parallel iso-frequency laminae; theoretically, this architecture permits penetrating electrode arrays to achieve frequency encoding that is more physiologically congruent in the depth dimension compared to ABIs [34]. Predicated on this anatomical advantage, the team led by Lenarz at Hannover Medical School pioneered clinical trials for the AMI. They utilized a specially designed penetrating multi-contact electrode array inserted directly into the ICC. Early clinical reports by Lim et al. (2009) demonstrated that AMI recipients derived significant benefits in environmental sound recognition and lip-reading assistance, with pitch discrimination capabilities in specific frequency bands superior to those of traditional ABI patients, thereby validating the feasibility of encoding based on central laminar structures [35]. However, long-term follow-up by Lenarz et al. (2013) identified limitations in the current technology: existing single-shank electrodes struggle to cover a sufficiently wide range of frequency laminae and fail to effectively simulate the complex temporal synchronization signals intrinsic to the IC, resulting in persistently low open-set speech recognition rates [36]. Current optimization efforts are primarily focused on the development of multi-shank arrays to expand coverage and the refinement of stimulation strategies to biomimetically replicate midbrain neural encoding patterns.

#### 2.3.2. High-Order Perceptual Reconstruction via Auditory Cortical Interfaces

The Auditory Cortex (AC) represents the highest center for processing complex acoustic features (e.g., language, music, spatial localization) and is simultaneously the genesis of pathological perceptions such as tinnitus. Research focus regarding cortical interfaces has shifted from early “simple sound evocation” toward “high-throughput decoding” and “minimally invasive implantation”.

The auditory cortex is not a homogeneous structure but comprises the primary auditory cortex (A1) and surrounding association cortices, responsible for spectral feature extraction and complex semantic processing, respectively. Contemporary research places increased emphasis on the neuromodulation functions of cortical interfaces. Studies by De Ridder et al. (2011) have indicated that intractable tinnitus is associated with pathological Gamma oscillations in the auditory cortex and paralaminar systems; interfering with this abnormal synchronized activity via cortical electrical stimulation has been observed to alleviate symptoms in certain drug-resistant tinnitus patients [37]. This suggests that ACIs possess a dual potential: serving as both a “write-in node” for auditory input and a “modulation node” for pathological states.

Cortical interfaces have long been constrained by the trade-off between surgical trauma and signal resolution: traditional Electrocorticography (ECoG) grids necessitate extensive craniotomy, while micro-needle arrays (e.g., the Utah Array), despite their high precision, offer limited coverage and induce brain tissue damage. Addressing this bottleneck, Zou et al. (2025), in a recent preprint, demonstrated a breakthrough “Guidewire-driven Deployable ECoG” system [38]. The team developed an ultra-flexible thin-film electrode array which, despite a thickness of only 650 nanometers, integrates 256 high-density channels within an effective area of 4 cm^2^.

The core innovation by Zou et al. lies in the utilization of a shape memory alloy guidewire to push the folded electrode array through a minute cranial burr hole (only a few millimeters in diameter) into the epidural space, where it automatically deploys and conformally adheres to the brain surface. In auditory testing using a canine model, the system successfully captured high-quality Auditory Evoked Potentials (AEPs) and clearly resolved neural response features to distinct sound stimuli [38]. This work not only resolves the issue of high invasiveness associated with traditional cortical implants but also provides unprecedented tools for decoding complex auditory cortical encoding mechanisms via its high channel density, making the future construction of minimally invasive, closed-loop systems for auditory reconstruction and tinnitus treatment viable.

### 2.4. Abnormal Neural Plasticity Induced by Auditory Deprivation and Its Modulation

Regardless of whether the interface targets the midbrain or the cortex, invasive implants fundamentally provide only exogenous electrical signal input. To achieve high-quality auditory rehabilitation, the brain must adapt to these artificial signals via synaptic plasticity mechanisms while suppressing maladaptive neural networks induced by chronic auditory deprivation (Figure 1b). Consequently, leveraging non-invasive or minimally invasive modalities to elicit endogenous neural plasticity has become a critical supplementary strategy for optimizing implantation outcomes and realizing neural rehabilitation.

Within the overarching architecture of auditory reconstruction brain–computer interfaces, mere signal input is often insufficient to address the complex neuropathological issues accompanying sensorineural hearing loss. Epidemiological data indicate that approximately 70% to 85% of patients with profound sensorineural hearing loss suffer from severe tinnitus. This comorbidity stems from maladaptive plasticity within the central nervous system: following cochlear damage and the consequent paucity of peripheral input, the auditory center attempts to “amplify” residual signals by downregulating inhibitory neurotransmitters (GABA) and upregulating excitability. While this mechanism maintains auditory sensitivity in the short term, chronic excessive gain leads to pathological spontaneous synchronized firing in the auditory cortex, termed “Thalamocortical Dysrhythmia (TCD)” [39]. Therefore, introducing non-invasive or minimally invasive adjunctive neuromodulation to suppress this aberrant activity and remodel erroneous network connectivity resulting from auditory deprivation constitutes a pivotal supplement for achieving high-quality auditory rehabilitation (Figure 1c).

#### 2.4.1. Transcranial Magnetic Stimulation (TMS) Modulation

Repetitive Transcranial Magnetic Stimulation (rTMS) represents the most extensively studied non-invasive modulation modality to date. Early strategies, predicated on the principle of “lateral inhibition”, primarily utilized low-frequency (1 Hz) stimulation of the auditory cortex (Heschl’s gyrus) to attenuate its hyperexcitability. However, Cima et al. [40] noted in the European multidisciplinary guideline for tinnitus that while traditional rTMS is effective for some patients, individual variability remains substantial, largely because tinnitus involves not only the auditory cortex but also emotional–cognitive networks encompassing the limbic system (e.g., amygdala and hippocampus).

Research trends over the past five years have shifted from “single-target” to “network-targeted” approaches. Lefaucheur et al. [41] highlighted in their meta-analysis that targeting the dorsolateral prefrontal cortex (DLPFC) with high-frequency stimulation, in conjunction with low-frequency stimulation of the auditory cortex, proves significantly more effective than modulating a single cortical site. The underlying mechanism is that the DLPFC, functioning as a cognitive control center, modulates the limbic system’s “gating mechanism” for tinnitus signals, thereby alleviating patient distress rather than merely reducing loudness [41]. Furthermore, personalized TMS (Navigated rTMS) based on resting-state functional MRI (rs-fMRI) localization is emerging as a new standard. Research by Seung Yeon Jeon et al. (2023) confirmed that utilizing imaging data to precisely target patient-specific hyperactive regions of the auditory cortex for stimulation improves therapeutic efficacy by approximately 30% compared to traditional anatomical localization [42].

#### 2.4.2. Vagus Nerve Stimulation (VNS) and Bimodal Modulation

Unlike TMS, which directly interferes with neural activity, Vagus Nerve Stimulation (VNS) aims to drive synaptic plasticity by activating neuromodulators, thereby “rewriting” the abnormal map of the auditory cortex. This approach largely benefits from breakthroughs in the theory of Bimodal Neuromodulation.

The seminal work by Engineer et al. [43] demonstrated that sound stimulation alone is insufficient to reverse cortical remodeling. However, when sound stimulation is paired with VNS, the cholinergic system of the basal forebrain and the noradrenergic system of the locus coeruleus, activated by the vagus nerve, provide a “learning signal” to the brain. This promotes the re-tuning of auditory cortical neurons to normal frequency sounds and suppresses abnormal synchronization at tinnitus frequencies [43]. Based on this principle, De Ridder et al. [44] tested implantable VNS paired with tone therapy in clinical trials, verifying its safety and potential efficacy. The team led by Susan Shore developed a more cutting-edge, non-invasive bimodal somatosensory stimulation technique. This method utilizes electrical stimulation of the trigeminal nerve (tongue or face) to simulate somatosensory input, synchronized with specific sound stimulation within a millisecond-level time window. This combined “auditory-somatosensory” stimulation leverages the multisensory integration mechanism of the dorsal cochlear nucleus (DCN) to induce Long-Term Depression (LTD), thereby suppressing the neural circuitry generating tinnitus at its source. Their large-scale double-blind randomized controlled trial published in JAMA Network Open showed that this method exhibits significant and durable efficacy in alleviating tinnitus loudness and distress, providing a potent non-surgical adjunctive modality for auditory rehabilitation [45,46].

## 3. Bio-Integration of the Neural Interface for Auditory Reconstruction BCIs

The long-term efficacy of auditory reconstruction BCIs is fundamentally contingent upon the degree of integration between abiotic devices and biological tissues at the microscopic interface. Traditional neuroprostheses, such as early wire ABIs or silicon-based Utah arrays, are constrained by a modulus mismatch of up to six orders of magnitude between their high Young’s modulus (~100 GPa) and that of brain tissue (~kPa level). This mechanical conflict not only provokes chronic foreign body response (FBR) and glial scar hyperplasia but also serves as the core etiology for long-term signal attenuation [47]. Recent research indicates that evolution in materials science is attempting to reconstruct the physicochemical properties of this interface through two dimensions: “substrate flexibilization” and “coating nanostructurization”.

### 3.1. Mechanical Matching and Biochemical Functionalization of Interface Materials

Material design for BCI devices is undergoing a transformation from singular electrical signal transmission toward multifunctional bioelectronic interfaces. Sahasrabudhe et al., in a 2025 review, pointed out that the next generation of bioelectronic interfaces requires transcending mere recording and stimulation functions, evolving toward multifunctional bioelectronics capable of simultaneous electrical, optical, and chemical interactions [48]. For auditory reconstruction BCIs, this implies that electrode materials must not only possess superior electrochemical properties to transmit micro-scale auditory encoding but also achieve deep integration with neural tissue at mechanical and biochemical levels, thereby establishing a long-term stable brain–computer loop.

#### 3.1.1. Flexible Substrates and Modulus Matching

To alleviate mechanical compression of neural tissue by implants, the morphological evolution of polymeric substrates has primarily revolved around stiffness reduction. However, a critical bottleneck in current auditory neuroelectrode development is the profound physical chasm between the Young’s modulus of rigid materials (e.g., silicon ~150 GPa, metals ~100 GPa) and that of soft neural structures like the cochlear nucleus, which typically ranges from 1 to 10 kPa [49]. This extreme modulus mismatch, often reaching six orders of magnitude, generates chronic shear stress and strain at the bio-interface, triggering glial scarring, chronic inflammation, and long-term signal attenuation [50].

Addressing the stringent requirements for tissue matching in auditory reconstruction, several feasible solutions have emerged. Regarding morphological optimization, Tybrandt et al. noted that reducing polymer thickness to the micrometer scale can cubically decrease bending stiffness and significantly enhance macroscopic conformability [51]. Recent work by Zou et al. validated this pathway by utilizing shape-memory alloy guidewires to minimally invasively deploy ultra-thin, folded Parylene/PI electrodes over extensive cortical areas [38]. From the perspective of material evolution, hydrogel-based hybrid interfaces are regarded as ideal candidates due to their high water content and tissue-like modulus (in the kPa range). These interfaces not only facilitate efficient ion-electronic signal transduction but also provide a microenvironment similar to the extracellular matrix. To balance surgical operability with long-term biocompatibility, shape-memory polymers (SMPs) offer a dynamic strategy; they maintain sufficient rigidity (GPa range) at room temperature to withstand insertion forces but rapidly soften to a compliant state (MPa range) once triggered by body temperature (37 °C) post-implantation [52]. Finally, as demonstrated by the soft Auditory Brainstem Implant (ABI) developed by Trouillet et al., leveraging the low bending stiffness of a 150-μm-thick silicone substrate allows for seamless conformal contact with the highly curved surface of the cochlear nucleus. By eliminating gaps between the electrode and the target tissue, such designs significantly reduce stimulation thresholds and enhance the spatial resolution of auditory percepts [33].

#### 3.1.2. Nanomaterial Interface Modification and Biochemical Functionalization

As auditory reconstruction devices evolve toward high channel counts and high density, the reduction in geometric surface area (GSA) leads to increased impedance and diminished charge injection capacity (CIC), presenting major physical limitations. An ideal auditory interface must possess low complex impedance for precise neural encoding and high charge storage capacity (CSC) for effective neuromodulation [53].

Regarding electrical performance, clinical standards such as platinum–iridium (Pt-Ir) alloys provide excellent biostability but suffer from limited charge transfer efficiency due to their smooth surfaces. Consequently, platinum black is frequently utilized to increase the effective surface area (ESA) through its fractal nanoparticulate morphology, thereby suppressing thermal noise [54]. Furthermore, iridium oxide (IrOx) is highly favored for high-frequency stimulation due to its rapid faradaic redox transitions involving Ir^3+^/Ir^4+^ states, which yield exceptional CSC [55]. In comparison, conductive polymers like PEDOT:PSS employ a volumetric transmission mechanism to extend electrochemical active sites into three-dimensional space, providing superior CIC even at miniaturized scales. To address the delamination of PEDOT coatings under chronic stimulation, current research focuses on enhancing interface adhesion by constructing interpenetrating networks or incorporating carbon nanotube (CNT) scaffolds to ensure electrochemical stability during long-term implantation (Table 1).

In terms of bioactivity, material design is increasingly incorporating biochemical modulation. Beyond leveraging graphene’s high specific surface area to promote synaptic adhesion, strategies have been established to utilize conductive polymers as “drug gates” [63,64]. These systems facilitate the on-demand release of anti-inflammatory drugs, such as dexamethasone, by disrupting electrostatic balances through electrochemical reduction [48]. This “chemo-actuation” design, integrating signal transmission with targeted therapy, represents a transition of electrodes from passive sensors to active therapeutic platforms capable of optimizing the implantation microenvironment by inhibiting gliosis.

### 3.2. Biomimetic Topological Structures and Ultra-High-Density Arrays

In addition to intrinsic material properties, the geometric topological structure of devices represents another critical pathway for resolving the mechanical mismatch between electrodes and neural tissue. Targeting the spiral structure of the cochlea and the complex curvilinear surfaces of the cochlear nucleus, traditional two-dimensional thin-film designs struggle to achieve intimate contact without inducing buckling or stress concentration. Recent research has shifted toward the design of mechanical metamaterial structures, such as serpentine and Kirigami patterns [65,66]. Optimized via finite element analysis, these biomimetic structures can convert tensile and bending stresses generated during implantation into the rotation and torsion of local beam structures, thereby achieving stress decoupling [67].

**Figure 2:**
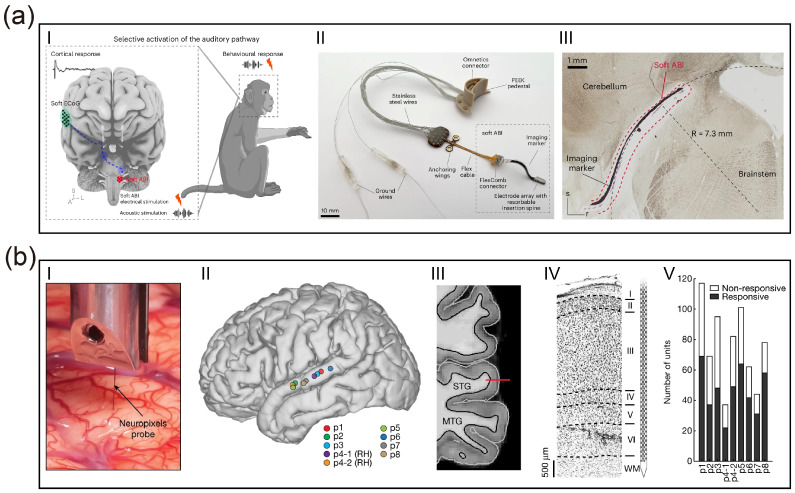
Advanced materials and biomimetic designs for auditory neural interfaces. (**a**) Validation of a soft ABI in non-human primates [33]. (I–II) Schematic of the closed-loop “brainstem stimulation–cortical recording” paradigm and photograph of the device featuring a resorbable spine. (III) Histological cross-section after 17 months of chronic implantation, demonstrating intimate conformal contact with the curved brainstem surface and long-term structural stability. (Creative Commons Attribution 4.0 International License). (**b**) High-resolution laminar recording in human auditory cortex using Neuropixels probes [68]. (I–IV) Intraoperative localization and histological verification of probe insertion in the Superior Temporal Gyrus (STG). (V) Distribution of speech-responsive single neurons across cortical layers, highlighting high-yield decoding capabilities. (Creative Commons Attribution 4.0 International License).

Advances in micro/nanofabrication processes have propelled electrode arrays into the thousand-channel scale, which is crucial for decoding complex auditory tonotopic maps. High density represents not merely a quantitative increase but a qualitative transformation in decoding mechanisms. Silicon-based probe technologies utilizing CMOS processes are continuously iterating toward higher densities and resolutions, driving auditory decoding from the level of local field potentials (LFPs) to single-unit resolution. Leonard et al. [68] applied high-channel-density Neuropixels probes to the superior temporal gyrus (STG) of human subjects for the first time, achieving large-scale recording of single-neuron firing activities across different depths of the cortical region [68] (Figure 2b). The study revealed that neurons within the auditory cortex exhibit significant laminar specificity in encoding vowels and consonants; such deep, fine spatiotemporal patterns are undetectable by traditional surface electrodes. This achievement demonstrates the immense potential of high-density intracortical microelectrodes in resolving complex speech features. Further demonstrating the high resolution of such high-density probes, Trautmann et al. utilized Neuropixels 1.0 and 2.0 probes in non-human primate models to achieve large-scale electrophysiological recordings across the whole brain. Their work maintained stable signal acquisition from thousands of channels over extended periods, specifically addressing issues of mechanical stability during probe insertion and signal drift in primate brains, where tissue volume far exceeds that of rodents [69]. Auditory reconstruction technologies based on CMOS ultra-high-density arrays, through an exponential increase in channel count at the hardware level combined with deep laminar decoding at the analytical level, hold promise for achieving high-fidelity reconstruction of complex auditory scenes.

### 3.3. Soft Actuation and Environmentally Adaptive Implantation Strategies

How to resolve the physical paradox of “implantation difficulty” while maintaining the low-trauma advantages of flexible electrodes constitutes a bottleneck in the engineering implementation of auditory BCIs. Traditional stylet-based implantation methods are prone to causing electrode recoil during guidewire removal, thereby damaging tissues surrounding the implantation target. Consequently, researchers have begun exploring “soft robotics-assisted” and “adaptive material” solutions.

Implantation strategies based on soft actuators offer a novel paradigm for the minimally invasive construction of cortical interfaces. A study published in Science Robotics by Song et al. (2023) proposed a soft electrode system based on an “eversion mechanism” [70]. Mimicking the growth patterns of plant roots, this technology utilizes fluid drive to extrude pre-folded microelectrode arrays—akin to unrolling a glove—through minute cranial burr holes, allowing for controlled deployment onto the cortical surface. This “self-actuating” mechanism not only circumvents the trauma associated with traditional large craniotomies but also achieves intimate coverage of large brain regions, such as the auditory cortex, without applying additional pressure, thereby resolving the challenge of implanting traditional flexible electrodes over extensive areas through minimally invasive apertures.

## 4. Neural Encoding Strategies and Computational Models for Auditory Reconstruction

The core challenge of auditory reconstruction BCIs lies not only in the expansion of hardware channels but, more critically, in transforming complex acoustic information into sequences of neural electrical impulses comprehensible to the brain. This process, termed “Sound Coding Strategy”, essentially establishes a mapping between the spectro-temporal features of external acoustic signals and the spatiotemporal stimulation patterns of the implanted electrode array. Despite breakthroughs in electrode materials and micro/nanofabrication technologies leading to an exponential increase in physical channel counts at the hardware level, current clinical coding strategies remain predominantly based on signal processing frameworks established decades ago. This strategic mismatch of “peripheralizing central hardware” prevents high-density arrays from fully leveraging their spatiotemporal resolution advantages within complex neural nuclei such as the brainstem or cortex. Consequently, this chapter explores the evolution of coding strategies from traditional waveform envelope extraction toward biomimetic computations adapted to distinct neuroanatomical levels—from cochlear tonotopy to cortical feature detection.

### 4.1. Traditional Envelope Coding and Its Limitations

The foundation for modern auditory prostheses was laid by the Continuous Interleaved Sampling (CIS) strategy proposed by Wilson et al. [14] in 1991. Prior to CIS, multi-channel analog stimulation frequently caused severe signal distortion due to the summation effects of simultaneous current fields, a particularly fatal issue within the narrow, fluid-filled environment of the cochlea. The core breakthrough of the CIS strategy was the introduction of an “asynchronous interleaving” mechanism. By decomposing full-band sound via a bank of bandpass filters, extracting the instantaneous envelope of each band using the Hilbert transform, and modulating the amplitude of these envelopes onto high-frequency biphasic pulse trains, CIS strictly controlled the timing of pulse delivery across channels. This ensured that only one electrode was active at any given instant, thereby effectively suppressing electric field crosstalk (Channel Interaction) between adjacent electrodes [14]. Although initially designed for the cochlear nerve, the lack of dedicated algorithms for the central nervous system led to the direct transplantation of CIS into ABI systems, where it remains the “gold standard” algorithm in auditory reconstruction.

To further reduce power consumption and minimize channel masking effects, researchers developed “N-of-M” type strategies based on CIS, the most representative being the Advanced Combination Encoder (ACE) employed by Cochlear Ltd. Unlike CIS, which activates all electrodes in every stimulation cycle, ACE utilizes Fast Fourier Transform (FFT) to analyze the spectrum in real-time, selecting only the channels with the highest energy (typically 8 to 12) for stimulation while suppressing lower-energy channels. This “maxima selection” mechanism leverages the psychoacoustic masking effect of the auditory system, in which the human ear tends to perceive strong signals while ignoring weak ones. The ACE strategy significantly reduces the total stimulation rate and system power consumption while preserving principal spectral information, improving speech intelligibility in quiet environments to a certain extent [71].

Despite the commercial success of CIS and ACE strategies in cochlear implants, their performance bottlenecks become increasingly apparent when applied to high-density arrays or extended to central targets (brainstem, cortex). These limitations manifest primarily as “physiology–algorithm” mismatches in both temporal and spatial dimensions.

**Figure 3:**
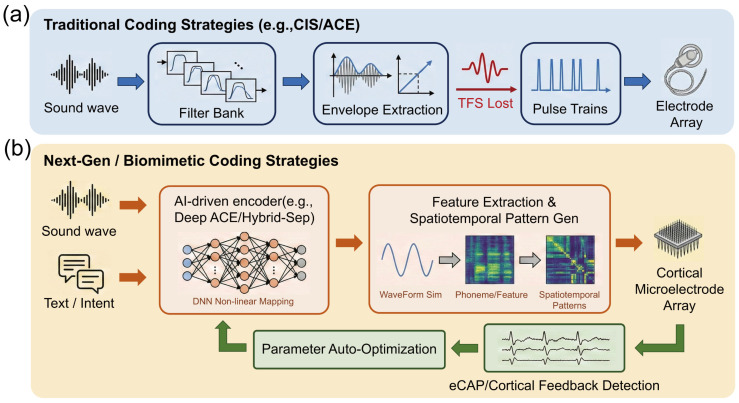
Paradigm shift in neural coding strategies for auditory prostheses: From linear envelope extraction to closed-loop biomimetic computation. (**a**) Traditional open-loop coding strategies (e.g., CIS/ACE). The acoustic input is processed through a linear filter bank, where only the temporal envelopes are extracted from varying frequency channels, resulting in the loss of temporal fine structure (TFS) information (indicated by the dashed internal waveforms). The final output consists of fixed, equidistant electrical pulse trains delivered uni-directionally to a standard cochlear implant electrode array. (**b**) Next-generation closed-loop biomimetic strategies. Multimodal inputs, combining acoustic signals with semantic intent signals (e.g., via brain–computer interfaces), are fed into an AI processing core (e.g., deep neural networks) for non-linear mapping. Instead of simple pulse trains, the system generates complex spatiotemporal activation patterns, depicted as a heatmap grid, which are written onto a high-density cortical microelectrode array. A crucial feedback loop utilizes physiological signals, such as electrically evoked compound action potentials (eCAPs) or auditory evoked potentials (AEPs), to enable real-time parameter auto-optimization, forming a bidirectional adaptive system.

First is the systematic absence of Temporal Fine Structure (TFS) (Figure 3a). Traditional strategies are fundamentally based on “envelope” coding; to prevent aliasing, low-pass filtering eliminates rapid phase change information above a few hundred Hertz. Research by Zeng Fan-Gang et al. indicates that TFS is crucial for pitch perception, sound localization, and separating target speech in noisy environments. For users of tonal languages such as Mandarin Chinese, the lack of TFS makes it difficult to identify the four tones via fundamental frequency (F0) variations. This issue is particularly acute in auditory brainstem implants because the cochlear nucleus processes temporal information via mechanisms more complex than the peripheral auditory nerve; simple envelope stimulation fails to elicit neural synchronization characteristic of natural hearing [72].

Second is the severe mismatch between spatial resolution and anatomical tonotopy. Traditional strategies assume a linear correspondence between electrode position and neuronal frequency tonotopy. While relatively valid in the cochlea, this assumption is challenged in central nuclei. In ABI, the frequency distribution on the surface of the cochlear nucleus is distorted and variable; traditional one-dimensional linear spectral mapping often leads to disordered pitch perception. In cortical auditory reconstruction, although high-density arrays offer physical channels at the micrometer scale, the broad current fields generated by traditional monopolar stimulation result in an effective number of channels far below the physical count. Classic studies by Shannon et al. demonstrated that under traditional coding, speech recognition rates saturate with 4 to 8 independent channels [73]. This implies that without altering the simple “position–frequency” mapping rules, merely increasing microelectrode density does not translate into improved perceptual resolution and may even deteriorate hearing due to exacerbated inter-channel interference.

### 4.2. Biomimetic Coding Based on Physiological Models

As auditory implant interfaces extend from the peripheral auditory nerve to central nuclei, a singular signal processing framework can no longer satisfy the physiological demands of different anatomical levels. The design of modern coding strategies is shifting from generic waveform envelope extraction toward differentiated computations based on “Anatomical-Functional Models”. Researchers have developed targeted spatiotemporal stimulation patterns tailored to the distinct neural coding characteristics of the cochlea, brainstem, and cortex.

#### 4.2.1. Current Focusing and Virtual Channels

At the level of CI, the core coding challenge lies in the stochastic firing of damaged neurons and current interference between electrodes. Addressing the restoration of neuronal physiological characteristics, Bruce et al. [74] refined the classic phenomenological model of the auditory nerve (Bruce–Zilany–Carney Model). This model quantitatively describes the neurotransmitter release kinetics between inner hair cells and auditory nerve synapses, precisely predicting the adaptation and refractory properties of auditory nerve fibers (ANF). This provides a benchmark for designing “biomimetic stimulation waveforms”, improving temporal information encoding efficiency by adjusting pulse interval statistics to simulate healthy spontaneous neural firing [74].

Addressing the current spread issue faced by high-density arrays, simply increasing physical contacts often fails due to “crosstalk”. Research by Bierer et al. [19] confirmed that adopting multipolar Current Focusing is key to resolving this. By applying anti-phase compensation currents on flanking electrodes (e.g., Tripolar mode), the excitatory electric field can be confined to a narrower cochlear region. Although this increases voltage requirements, focusing strategies significantly improved Spectral Modulation Ripple Thresholds (SMRT) in clinical trials, proving that enhanced spatial selectivity is superior to mere loudness growth [19]. Furthermore, the classic virtual channel technology proposed by Firszt et al. [75] remains a core method for enhancing frequency resolution. By proportionally distributing current between adjacent electrodes, it successfully induces “intermediate pitch” percepts between two physical contacts, which are crucial for music melody appreciation [75].

#### 4.2.2. Sparse Coding Strategies and Effective Site Selection

When auditory reconstruction advances to the brainstem (ABI) level, the primary obstacles are: first, the current spread generated by electrical stimulation in highly conductive brainstem tissue easily leads to non-specific activation and crosstalk between adjacent frequency channels; second, the tonotopy on the surface of the cochlear nucleus is far more complex than that of the cochlea, exhibiting distorted and fragmented characteristics that render traditional linear coding strategies ineffective.

Within the existing electrical stimulation framework, Trouillet et al. [33] proposed a “Sparse Efficient Coding” strategy combining computational modeling and machine learning to address the complex tonotopy of the brainstem surface. They recognized that in high-density arrays (e.g., 32 channels or more) covering the brainstem, not all contacts elicit effective auditory perception; indiscriminate full-array stimulation only introduces background noise. Consequently, the team established a closed-loop screening process: first using Finite Element Models (FEM) to predict current field distribution after soft electrode contact with the brainstem surface; then employing machine learning algorithms (e.g., Gaussian Process Classifiers) to analyze cortical responses and identify an “Effective Subset” of physical channels capable of inducing clear, independent auditory cortical responses. By discarding ineffective redundant channels and utilizing only the screened sparse sites for precise stimulation, this strategy successfully reconstructed highly frequency-specific cortical activation maps in non-human primate models, demonstrating that redefining the electrode–neural mapping via “computational screening” is essential to truly unlocking the hardware potential of high-density ABIs [33].

#### 4.2.3. Feature-Driven Cortical Stimulation and Perceptual Reconstruction

At the level of the Auditory Cortex (AMI/ACI), coding strategies completely abandon the simulation of peripheral waveforms, shifting toward the direct inscription of “Percepts”. Cortical neurons are primarily sensitive to sound features (e.g., onset/offset, modulation direction, phoneme category) rather than sound pressure waveforms. Beauchamp et al. (2020) established the principle of Dynamic Current Steering in research on visual cortical prostheses, a principle equally applicable to the auditory cortex. They found that static electric fields applied to the cortical surface often lead to rapid perceptual adaptation, whereas rapidly moving the current center along specific trajectories across the electrode array not only sustains perception but can also encode spatial information via scan direction [76]. Regarding high-level language encoding in the human auditory cortex, Recent research by Leonard et al. [68] revealed that neurons in the superior temporal gyrus exhibit deep laminar specificity for encoding consonants and vowels. This suggests that future cortical coding strategies should be “Feature-driven”: utilizing deep learning models to extract phonemic features from speech and then activating corresponding cortical columns via high-density microelectrode arrays according to specific spatiotemporal patterns, rather than simply “playing” sound waveforms [68].

### 4.3. Closed-Loop Control and AI Augmentation

Traditional “open-loop” coding strategies, once parameterized, do not adapt to dynamic changes in the implantation environment or complex neural topologies. To overcome this, modern coding systems are developing differentiated closed-loop and intelligent optimization schemes depending on the interface location (Figure 3b).

#### 4.3.1. Electrophysiological Closed-Loop Feedback Mechanisms

At the Cochlear Implant (CI) level, the primary goal of closed-loop control is to resolve physical coupling issues at the electrode–neural interface (e.g., impedance fluctuation, current spread). As auditory nerve fibers are relatively concentrated, the electrically evoked Compound Action Potential (eCAP) provides a stable, high signal-to-noise ratio feedback signal, making it the core of CI closed-loop systems. Addressing current field overlap within the cochlea, Goehring et al. [77] proposed Panoramic eCAP technology. This method uses the CI to reverse-telemetry neural response potentials across the full array, calculating the patient-specific Current Spread coefficient in real-time. When the system detects excessive current spread in a channel causing non-specific excitation of adjacent neurons, it automatically triggers a “Focusing” compensation mechanism or adjusts stimulation rates, thereby maintaining optimal spatial resolution without interrupting auditory input [77]. According to the framework discussed by Van Opstal and Noordanus [78], Gaussian Process-based Bayesian Optimization has been proposed to automate the fitting process. This system utilizes eCAP as objective physiological feedback combined with simple binary user evaluations of sound quality (“better” or “worse”) to automatically explore the vast parameter space (e.g., pulse width, threshold levels) via probabilistic models. Experiments showed that the algorithm converges to personalized optimal parameter sets within very few interaction steps, significantly improving fitting efficiency and providing precise parameter configuration solutions for infants or cognitively impaired patients unable to provide clear verbal feedback [78].

#### 4.3.2. Deep Learning-Driven Non-Linear Mapping

For ABI and AMI, the core challenge is not merely current spread but severe frequency tonotopic distortion. Traditional filter-bank strategies assume a linear “position–frequency” relationship, which fails completely in central nuclei with disordered topology. Optimization at this level therefore relies on the “end-to-end” non-linear mapping capabilities of deep learning. The Deep ACE model proposed by Gajecki et al. [79] provides a general framework for this problem. This model uses Deep Neural Networks (DNN) to directly establish a mapping from “acoustic waveforms” to “electrical stimulation patterns” [79]. Although initially validated in CIs, its “learnable” nature makes it particularly critical for ABIs: AI models can be trained to automatically learn how to assign sound features to non-linearly distributed effective electrodes on the brainstem surface, bypassing topological mapping difficulties intractable for traditional DSP algorithms. Furthermore, addressing the lack of central integration in Bilateral Implants, Gajecki et al. [80]) proposed Fused Deep Coding. By fusing information from left and right ears within the neural network’s Latent Space, they successfully reconstructed Interaural Time Difference (ITD) cues. For ABI/AMI patients lacking natural binaural neural pathways, this represents a key algorithmic breakthrough for restoring sound localization capabilities [80].

#### 4.3.3. Semantic Understanding and Active Auditory Attention

Regardless of whether the implant is cochlear or central, the “Cocktail Party Effect” (attending to a specific speaker in a noisy environment) is a common difficulty for all auditory prosthesis users. Closed-loop control at this level relies not on local neural feedback from the implant site but on high-level intent signals from the brain to modulate front-end sound processing. Geirnaert et al. [81] demonstrated the feasibility of using circum-aural EEG for general Auditory Attention Decoding (AAD). Whether the back-end is a CI or ABI, the system can identify the user’s current attentional target by analyzing cortical tracking responses to different sound source envelopes, instructing the front-end microphone array to directionally enhance that target speech [81]. Feng et al. [82], in their latest work on Hybrid-Sep, demonstrated breakthrough progress in Language-Query Audio Source Separation (LASS). Unlike passive gaze tracking, this technology allows users to control front-end processing via natural language commands (e.g., “I want to hear the piano clearly” or “Enhance the male voice”). The Hybrid-Sep model fuses Contrastive Language-Audio Pre-training (CLAP) with Self-Supervised Learning (SSL) models, utilizing Adversarial Diffusion Training to precisely isolate target objects from complex mixed acoustic scenes based on user semantic descriptions. This mechanism endows auditory reconstruction systems with unprecedented semantic understanding capabilities, transforming users from passive signal receivers into active constructors of their auditory scenes [82].

## 5. Future Prospects

### 5.1. Frontier Technologies for High Spatiotemporal Resolution

**Figure 4:**
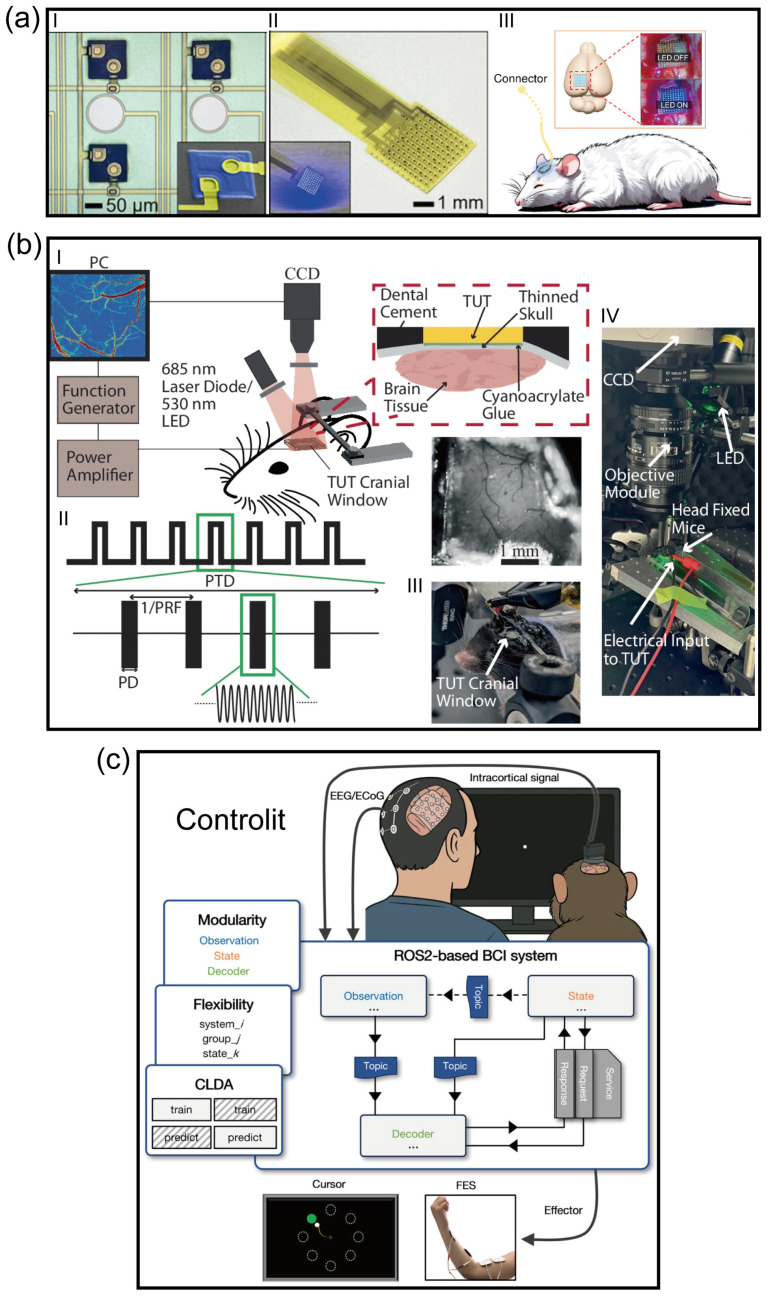
High-spatiotemporal-resolution neuromodulation technologies and a universal control architecture. (**a**) Monolithic optoelectronic array for artifact-free cortical optogenetics [84]. (I–II) Characterization of the integrated interface combining high-density micro-LEDs with ECoG electrodes. A localized mean subtraction algorithm is employed to suppress photoelectric artifacts, ensuring high-SNR recording. (III) Schematic of the device implanted on the rat cortex for precise optical stimulation. (Copyright 2024, American Chemical Society. Reproduced with permission). (**b**) Transcranial ultrasound neuromodulation with concurrent optical imaging [85]. (I) Experimental setup for awake mice utilizing a Transparent Ultrasound Transducer (TUT) to enable simultaneous deep-brain modulation and cortical imaging through a thinned skull. (II–IV) Stimulation protocols and photographs of the head-fixed apparatus validating system stability. (Copyright 2026, The Author(s). Reproduced with permission). (**c**) “ControlIt” universal BCI framework [91]. Schematic of the ROS2-based architecture designed to unify heterogeneous neural data streams. The framework features a modular design (decoupling Observation, State, and Decoder) and supports Closed-Loop Decoder Adaptation (CLDA) for real-time algorithm updates and low-latency transmission. (CC BY 4.0).

Despite the establishment of electrical stimulation as the clinical “gold standard” for artificial auditory implants over the past few decades, its intrinsic physical limitations have become increasingly prominent. Due to the passive diffusion effect of electrical currents in biological tissues, existing neural prostheses struggle to avoid channel crosstalk, resulting in a spectral resolution bottleneck that is physically insurmountable. Addressing this core challenge, Keppeler et al. [83] proposed a solution based on optogenetics. Leveraging the extreme spatial focusing capability of photons, they successfully confined neural excitation to ultra-narrow regions by expressing light-sensitive proteins (Channelrhodopsin) in auditory neurons, providing a theoretical basis for achieving frequency resolution approaching physiological levels [83]. To further meet the hardware demands for high-density modulation, Gu et al. [84] developed a large-scale optogenetic device integrating hundreds of micro-LEDs with ECoG electrodes (Figure 4a). This system not only achieved pixel-level precise activation of neuronal populations through customized control but also overcame the challenge of photoelectric artifacts using a local average subtraction algorithm, realizing high signal-to-noise ratio in situ neural recording while maintaining low thermal effects [84]. However, while optogenetics resolves the “lateral” spatial resolution issue, it is limited by the scattering and attenuation of light waves in tissue, making it difficult to reach deep auditory centers such as the cochlear nucleus or inferior colliculus. Furthermore, the thermal effects associated with implantable light sources and the complexity of packaging limit the stability of its long-term application.

To overcome the depth limitations of photons while maintaining high-precision modulation, recent research has turned to ultrasound stimulation, particularly the “sonogenetics” strategy combined with genetic engineering. As demonstrated by recent studies, ultrasound possesses excellent physical properties for penetrating bone and soft tissue, reaching deep neural nuclei with minimal attenuation. Through focused ultrasound (FUS) technology or by utilizing mechanosensitive ion channels (such as MscL, Piezo1, or newly discovered ultrasound-sensitive proteins), researchers can achieve sub-millimeter precision activation of specific neural circuits without compromising tissue integrity [85] (Figure 4b). This “sound-controlled” mode not only avoids the invasive damage of optical fiber implantation but also theoretically provides a universal physical means for a fully implantable, deep-probe-free auditory brain–computer interface capable of covering the entire auditory pathway (from cochlea to cortex).

However, the practical application of ultrasound stimulation in auditory reconstruction faces severe challenges regarding temporal resolution and energy transduction. First, the auditory system requires extremely high standards for encoding Temporal Fine Structure (TFS) (microsecond to millisecond scale), whereas existing ultrasonic neuromodulation mechanisms often involve mechanical force transduction or micro-thermal effects, resulting in neural response latencies and recovery cycles significantly longer than those of electrical or optical stimulation. This implies that ultrasound may struggle to perfectly encode high-frequency speech envelope information, potentially causing patients to “hear sound” but fail to “distinguish semantics.” Second, despite its strong penetration, ultrasound poses potential biological safety risks due to reflection at the cranial interface and cavitation effects at the microscopic scale. Additionally, similar to optogenetics, sonogenetics relies on viral vectors for gene delivery; its transfection efficiency, immune response, and the stability of long-term expression in primates and humans constitute major biological barriers to clinical translation.

Looking ahead, the trend for clinical transition will be characterized by non-viral approaches and multimodal fusion. On one hand, to circumvent the risks of gene therapy, research focus is gradually shifting from virus-mediated sonosensitive proteins to inorganic nanotransducers (such as piezoelectric nanoparticles). These materials can directly convert ultrasound waves into local electric fields to stimulate nerves, combining the penetration depth of ultrasound with the rapid response of electrical stimulation, while offering better biosafety. On the other hand, before single-modal technologies mature, a “Light-Sound-Electricity” hybrid strategy may serve as a transitional solution: utilizing electrical stimulation to provide high temporal resolution loudness envelopes, employing optical or ultrasonic stimulation to deliver high spatial resolution spectral information, or using ultrasound for coarse modulation of deep nuclei combined with surface electrodes for fine coding. This engineering compromise may represent the most viable path connecting fundamental biophysical research with clinical auditory rehabilitation applications.

### 5.2. Dynamic Topological Reconfiguration and Developmentally Driven Symbiotic Integration

Existing neural interface research largely focuses on passively adapting to the biological environment via material softening (as discussed in Section 3). However, this static compatibility strategy cannot fundamentally eliminate the chasm between abiotic devices and dynamic biological tissues. To achieve ultimate long-term stability and high resolution, future auditory reconstruction technologies must undergo a fundamental paradigm shift: from static physical replacement to intelligent systems capable of dynamic topological reconfiguration and active symbiotic behavior.

#### 5.2.1. Dimensional Reduction and Topological Reconfiguration: Rolling Spirals and High-Density Integration

Traditional micro-nanofabrication technologies are constrained by two-dimensional (2D) planes, making it difficult to achieve extremely high channel density while maintaining minimally invasive dimensions. Addressing this contradiction, Khatib et al. (2025) proposed a manufacturing paradigm of “Dimensional Reduction”, wherein high-precision multimodal sensor arrays are fabricated on a 2D plane and subsequently rolled into a one-dimensional “Spiral NeuroString” utilizing stress-release mechanisms [86]. This “rolled-up” topological reconfiguration strategy offers dual advantages. First, it compresses a centimeter-scale planar functional area into a micron-scale fiber cross-section, realizing for the first time the integration of over 1000 multimodal channels (electrophysiology, neurotransmitters, optogenetics) on a single flexible fiber, vastly enhancing the information throughput of auditory decoding. Second, its unique spiral topology endows the device with excellent axial extensibility and radial compliance, allowing it to buffer physiological micromotion within the brainstem or cochlea like a spring, thereby eliminating mechanical abrasion during long-term implantation. For Auditory Brainstem Implants (ABI), this structure implies that electrodes can conformally adhere to the complex curvature of the brainstem surface, resolving the contact issues of traditional flat electrodes.

#### 5.2.2. Active Bioelectronics with Adaptive Motility for Dynamic Interfacing

Implantable electrodes have long been viewed as “foreign bodies” requiring physical anchoring, with electrode drift caused by tissue micromotion being a primary cause of signal decay. Xie et al. (2025) upended this perception by developing a “Movable Soft Microfibre” named NeuroWorm, inspired by earthworm locomotion mechanisms [87]. This device is no longer a passively fixed probe but an intelligent agent with active motility. Utilizing magnetic drive and a segmented structural design, NeuroWorm can perform micron-level precise “swimming” or position reconfiguration within brain tissue after implantation. This characteristic brings revolutionary possibilities to auditory reconstruction: the implant can actively search for and lock onto the optimal “hotspots” of auditory neurons post-surgery, or even perform adaptive positional adjustments in response to patient growth or anatomical changes in brain tissue, effectively circumventing functional failure caused by electrode migration and realizing true “lifelong in vivo dynamic tracking”.

#### 5.2.3. Developmentally Driven Symbiotic Integration

At a more extreme level of biological fusion, the intervention window of the future may shift significantly earlier. Sheng et al. (2025) demonstrated a “Symbiotic Development” strategy, utilizing the mechanical matching of ultra-soft mesh electrodes with embryonic neural plates to allow the device to be “woven” deep into the neural network by endogenous growth forces during neurulation [88]. This “grow first, transplant later” paradigm suggests that, combined with organoid technology, we might in the future cultivate “bio-hybrid cochleae” pre-embedded with high-density electrode arrays in vitro. These could be transplanted as complete auditory organs, thereby achieving an atomic-level seamless fusion of electronic devices and biological tissues.

### 5.3. AI-Assisted and Intent-Driven Intelligent Ecosystem Reconstruction

Future auditory reconstruction technology should not merely pursue the stacking of algorithms and computing power, but rather strive for a fundamental reconstruction of the interaction paradigm. Although current implants utilize noise reduction algorithms, their parameter fitting remains at the “static calibration” stage, unable to cope with dynamically changing neurophysiological states. Future technological development should focus on constructing unsupervised adaptive closed loops. This requires shifting research focus from singular auditory signal processing to the real-time mining of objective electrophysiological markers (e.g., eCAP, cortical evoked potentials). As demonstrated by Gao and Nogueira (2023), replacing traditional manual fitting with a Bayesian optimization framework is not merely for efficiency; its deeper value lies in establishing a “data-driven parameter adaptation standard” [78]. Future systems should possess the capability to continuously micro-adjust stimulation strategies based on neural feedback without user awareness, transforming the device from a “preset” tool into a “symbiont” that remodels itself alongside the user’s auditory pathway.

The deep integration of AI algorithms is evolving implants from passive signal converters into “auditory co-processors” equipped with computational intelligence. First, at the signal processing level, deep learning-based End-to-End algorithms are replacing traditional Digital Signal Processing (DSP) modules. Recent research shows that using advanced architectures like Deep Complex Convolutional Transformer Networks (DCCTN), AI models can precisely separate target speech from extremely noisy non-stationary environments (e.g., streets, restaurants), offering significant improvements in speech intelligibility over traditional noise reduction algorithms [11,89]. Furthermore, the “audio-to-electrical” direct mapping model proposed by Gajecki et al. allows AI to automatically learn how to convert acoustic features into optimal electrical pulse sequences, bypassing the inherent information loss in traditional envelope extraction [79]. Second, at the level of parameter fitting and personalized configuration, AI-driven “Digital Twin” technology is altering clinical pathways. By constructing patient-specific cochlear anatomical models via high-resolution CT imaging, AI algorithms can perform tens of thousands of stimulation strategy simulations and optimizations in a virtual environment to predict optimal electrode positioning and current parameters [90]. Combined with Reinforcement Learning, the system can continuously fine-tune stimulation strategies based on neural feedback (e.g., eCAP) without conscious user intervention.

Simultaneously, the ultimate goal of human–machine interaction should be “zero-load intent sensing”. Existing auxiliary functions (such as manual scene switching) consume excessive cognitive resources. The direction of technological development must be to endow implants with semantic-level scene understanding and cognitive state monitoring capabilities. Based on the Hybrid-Sep model proposed by Feng et al. (2025), we see the potential of using semantic instructions to control auditory filtering [82]. However, future evolution should further integrate multimodal sensors (such as eye-tracking or EEG Alpha wave monitoring) to assess the user’s “Listening Effort” and “attentional focus”. The system should not wait for user commands but should predict the user’s listening intent through multimodal fusion—for instance, automatically locking onto a sound source and suppressing background noise upon detecting the user gazing at a person with dilated pupils. This paradigm shift from “instruction control” to “intent prediction” is key to truly integrating auditory prostheses into the patient’s sensory experience.

To support this high-dimensional multimodal interaction and real-time adaptive evolution, the underlying software architecture must undergo a qualitative change from “specialized silos” to a “universal intelligent framework”. The ControlIt framework recently proposed by Yang et al. [91] provides a quintessential engineering example of this paradigm shift (Figure 4c). This study constructed a modular, universal brain–computer interface architecture based on the Robot Operating System (ROS 2), achieving for the first time the seamless system-level fusion of multimodal neural signals (covering non-invasive EEG to invasive ECoG and single-neuron spikes) with external environmental sensor data [91]. The core breakthrough of ControlIt lies in its “streaming-based” design philosophy and the deep integration of a Closed-loop Decoder Adaptation (CLDA) module. This enables auditory reconstruction systems not only to process heterogeneous signals from the cochlea, brainstem, and cortex in parallel but also to real-time update decoding algorithms to adapt to brain neuroplasticity without offline recalibration. This universal software infrastructure will be the key to building “bio-electronic symbionts” in the future, breaking down barriers between different implant modalities and providing a scalable computational foundation for cross-scale, multi-source information-driven intent-aware auditory ecosystems.

### 5.4. Challenges and Pathways for Clinical Translation

The transition of advanced auditory BCIs from laboratory prototypes to clinical reality necessitates addressing critical translational bottlenecks that extend beyond initial functional validation.

#### 5.4.1. Biocompatibility and Long-Term Interface Stability

The primary challenge in translating auditory BCIs lies in the chronic stability of the interface after implantation. A mechanical modulus mismatch that reaches six orders of magnitude between rigid abiotic devices and soft neural tissue is the core trigger for chronic foreign body responses (FBR). This persistent irritation leads to gliosis and the formation of non-conductive fibrous capsules. Such encapsulation increases electrode impedance, obstructs neurotransmitter diffusion, and expands the electrode-to-neuron distance. These factors collectively result in signal attenuation and a progressive increase in activation thresholds over time [47]. Future research must focus on leveraging ultra-flexible materials and bioactive “drug gates” such as dexamethasone-loaded polymers to mitigate these reactions and achieve true “bio-symbiotic” integration.

#### 5.4.2. Scalability of High-Density Micro-LED Arrays for Human Use

While miniaturized optoelectronic arrays have performed excellently in non-human primate experiments, scaling these technologies for human clinical use faces significant engineering hurdles. To accommodate human anatomy, arrays must achieve larger area coverage while maintaining ultra-flexibility through the use of 150-μm-thick silicone substrates or sub-micron thin-film electrodes [33]. This scale-up requires sophisticated microfabrication processes to maintain the interconnect integrity of thousands of channels. Furthermore, the development of highly integrated implantable application-specific integrated circuits (ASICs) is required to manage the wireless data bandwidth and power supply demands inherent in human-scale systems.

#### 5.4.3. Long-Term Safety of Chronic Photostimulation and Electrical Stimulation

In the pursuit of high spatial resolution, the safety of long-term physical intervention must be rigorously evaluated. For emerging optogenetics-mediated auditory restoration, the photothermal effects caused by chronic light stimulation are a primary concern because continuous micro-LED illumination may lead to localized temperature increases that damage neurons [83]. Strategies such as localized mean subtraction algorithms and optimized pulsed modulation are essential to maintain low thermal effects. Additionally, the reduction in microelectrode size for high-density electrical interfaces significantly increases local charge density [84]. It is critical to ensure that these levels remain within the electrochemical safety window to prevent electrode corrosion or electrolytic tissue damage [92]. Future clinical systems must integrate real-time closed-loop monitoring to ensure long-term biological safety.

### 5.5. Neuro-Privacy Protection and Technological Equity

At the therapeutic level, future development must break the traditional concept that “treatment ends with surgery” and shift towards constructing a “precision rehabilitation system driven by active neuromodulation”. Current rehabilitation models rely too heavily on passive patient adaptation, neglecting the active remodeling of peripheral-central neural circuits. The Shore team’s research on bimodal stimulation points the way: rehabilitation technology should evolve from general auditory training to precision intervention targeting specific windows of neuroplasticity [46]. Future implants should serve not only as ports for signal input but also as execution terminals for neuro-rehabilitation—specifically, inducing LTP or LTD through synergistic electrical stimulation patterns (such as paired pulse stimulation) within specific time windows to actively “erase” tinnitus networks or strengthen language center connectivity. This path of “medical–engineering combination” requires that future hardware designs reserve interfaces for bidirectional neuromodulation, allowing devices to dynamically adjust stimulation strategies according to rehabilitation progress, thereby breaking through the current plateau in speech recognition rates.

When auditory reconstruction technology begins to penetrate the central nervous system and introduces AI agents (like Hybrid-Sep) to intervene in the construction of perception, we must confront the ethical challenges it brings. The primary issue is Mental Privacy and data ownership. Next-generation implants with closed-loop recording capabilities will inevitably collect high-dimensional neural data containing emotions, attentional preferences, and even pathological features. The “Neurorights” framework proposed by Yuste et al. (2017) points out that legal boundaries must be established to prevent neural data from being commercially abused or used to build user profiles [93]. Examples include developing edge-side encryption technology to ensure neural data does not leave the device, and designing “Reality Penetration” modes to prevent information cocoon effects caused by excessive algorithmic filtering. Furthermore, when AI algorithms replace the brain in deciding “what sounds to filter”, the user’s personal Agency faces blurring: if an algorithm blocks certain environmental sounds based on semantic instructions, is the “reality” perceived by the user distorted by algorithmic bias? This “perceptual filtering” may have profound invisible impacts on individual social interactions.

With the introduction of enhanced functions (such as ultra-long-distance sound pickup, real-time language translation, and multimodal fusion), high-performance auditory BCIs may become extremely expensive. Ienca and Andorno (2017) warned that this could lead to social differentiation at the biological level, where the wealthy gain cognitive and perceptual abilities surpassing ordinary humans through technology, while vulnerable groups cannot even obtain basic hearing restoration [94]. Therefore, future engineering R&D must incorporate Accessibility as a core consideration, ensuring that neural interface technology serves as a bridge to close the disability gap, rather than a catalyst for exacerbating social inequality.

## 6. Conclusions

The developmental trajectory of auditory reconstruction BCIs represents, in essence, an engineering odyssey aimed at bridging pathological chasms and restoring sensory pathways. From the initial validation of electrical hearing by Djourno and Eyriès in 1957, to the establishment of cochlear implants as the clinical gold standard for peripheral substitution, and finally to the current advancement of ABIs and midbrain/cortical interfaces into the depths of the central nervous system, the evolution of this field clearly delineates a trend of ascending intervention targets. Throughout this review, the underlying tension identified is the persistent interplay between increasingly sophisticated engineering technologies and the complex, elusive biological characteristics of the central nervous system. Early endeavors were often constrained by a “confrontation” at the physical level, exemplified by mechanical mismatches and inflammatory responses induced by rigid electrodes. However, with deepening research, the technological trajectory has undergone a fundamental transformation, establishing an evolutionary paradigm shifting from “physical replacement” to “biological symbiosis”.

This fusion achieves a dialectical synthesis across both hardware and software dimensions. In the hardware domain, the introduction of ultra-flexible nanomaterials, shape-memory polymers, and “dimensional reduction” fabrication processes has finally endowed neural interfaces with the capability to achieve “zero-stress” conformal contact with brain tissue. This fundamentally mitigates the foreign body response associated with chronic implantation, laying the material foundation for long-term stable signal transmission. In the realm of software interaction, coding strategies have undergone a qualitative metamorphosis from generic linear waveform simulation to biomimetic feature writing. Confronted with the highly non-linear tonotopy of the brainstem and cortex, the limitations of traditional algorithms have necessitated the deep integration of artificial intelligence and deep learning technologies. Through the establishment of end-to-end non-linear mapping and closed-loop feedback mechanisms, modern implants are beginning to possess the capability to perceive neural states in real-time and dynamically optimize stimulation parameters, truly realizing the leap from unidirectional “open-loop stimulation” to bidirectional “intelligent interaction”.

In conclusion, auditory reconstruction brain–computer interfaces stand at a critical historical juncture: they are evolving from mere functional replacement tools into symbiotic systems capable of deep integration with biological neural networks. The ultimate value of this technology lies not only in restoring the world of sound for patients beyond the reach of traditional medical interventions (such as those with NF2 or cochlear nerve aplasia) but also in unlocking the possibility for humanity to reconstruct perception through engineering means. As interdisciplinary barriers between materials science, neural computing, and clinical medicine are further dismantled, we have reason to believe that future auditory prostheses will no longer be assistive devices for disability repair. Instead, they will serve as bridges connecting biological intelligence with artificial intelligence, reshaping the human sensory experience, and thereby—premised on ensuring ethics and equity—fundamentally transforming the destiny of the hearing-impaired population.

## Figures and Tables

**Table 1:** Comparative performance of functional nanomaterial-modified interfaces.

Modified Materials	Geometric Area [μmm^2^]	|Z| at 1 kHz [Ω·mm^2^]	CSC/CSCc [mC/cm^2^]	CIC/CIL [mC/cm^2^]	Reference
PtNPs	5000	390	1.2	2.67	[56,57]
IrOx	177	20.1	28.8	4.7	[54,55]
Pt-Ir	4417.8	50	12.5	0.15	[58]
PEDOT:PSS	177	4.1	75.6	8.5	[59]
PEDOT:CNT	706.5	10.95	7.5	6.5	[60]
PEDOT:PSS/PtNPs	706.5	7.73	14.84	4.37	[61]
PEDOT:PSS/AuNPs	706.5	8.61	3.05	5.7	[61]
PEDOT/3-MPA-Au	7850	31.71	7.21 (CSCc)	NA	[62]

## Data Availability

No new data were created or analyzed in this study. Data sharing is not applicable to this article.

## Abbreviations

The following abbreviations are used in this manuscript:

BCIs	Brain–Computer Interfaces
SNHL	Sensorineural Hearing Loss
WHO	World Health Organization
PAF	Population Attributable Fraction
CI	Cochlear Implant
NF2	Neurofibromatosis Type 2
ABI	the Auditory Brainstem Implant
AMI	Auditory Midbrain Implants
ACI	Auditory Cortical Interfaces
CIS	Continuous Interleaved Sampling
CPA	Cerebellopontine Angle
IC	Inferior Colliculus
ICC	the Central Nucleus of the Inferior Colliculus
AC	Auditory Cortex
A1	the Primary Auditory Cortex
ECoG	Electrocorticography
TCD	Thalamocortical Dysrhythmia
TMS	Transcranial Magnetic Stimulation
rTMS	Repetitive Transcranial Magnetic Stimulation
DLPFC	dorsolateral prefrontal cortex
VNS	Vagus Nerve Stimulation
DCN	Dorsal Cochlear Nucleus
LTD	Long-Term Depression
FBR	Foreign Body Response
LFPs	Local Field Potentials
STG	Superior Temporal Gyrus
SMP	Shape Memory Polymers
MRI	Magnetic Resonance Imaging
ACE	Advanced Combination Encoder
FFT	Fast Fourier Transform
TFS	Temporal Fine Structure
SMRT	Spectral Modulation Ripple Thresholds
ANF	Auditory Nerve Fibers
FEM	Finite Element Models
DNN	Deep Neural Networks
ITD	Interaural Time Difference
AAD	Auditory Attention Decoding
LASS	Language-Query Audio Source Separation
CLAP	Contrastive Language-Audio Pre-training
SSL	Self-Supervised Learning
eCAPs	evoked Compound Action Potentials
AEPs	Auditory Evoked Potentials
FUS	Focused Ultrasound
DSP	Digital Signal Processing
DCCTN	Deep Complex Convolutional Transformer Networks
CLDA	Closed-loop Decoder Adaptation
TUT	Transparent Ultrasound Transducer
PTD	Pulse Train Durations
PRF	Pulse Repetition Frequencies
